# Automatic Subsidence Troughs Detection in SAR Interferograms Using Circlet Transform

**DOI:** 10.3390/s21051706

**Published:** 2021-03-02

**Authors:** Justyna Bała, Maciej Dwornik, Anna Franczyk

**Affiliations:** Department of Geoinformatics and Applied Computer Science, AGH University of Science and Technology, al. Mickiewicza 30, 30-059 Kraków, Poland; jbala@agh.edu.pl (J.B.); dwornik@agh.edu.pl (M.D.)

**Keywords:** circlet transform, ellipse detection, subsidence troughs

## Abstract

This article presents the results of automatic detection of subsidence troughs in synthetic aperture radar (SAR) interferograms. The detection of subsidence troughs is based on the circlet transform, which is able to detect features with circular shapes. Compared to other methods of detecting circles, the circular transform takes into account the finite data frequency. Moreover, the search shape is not limited to a circle but identified on the basis of a certain width. This is especially important in the case of detection of subsidence troughs whose shapes may not be similar to circles or ellipses but to their fragments. The transformation works directly on the image gradient; it does not require further binary segmentation or edge detection as in the case of other methods, e.g., the Hough transform. The entire processing process can be automated to save time and increase reliability compared to traditional methods. The proposed automatic detection method was tested on a differential interferogram that was generated based on Sentinel-1A SAR images of the Upper Silesian Coal Basin area. The test carried out showed that the proposed method is 20% more effective in detecting troughs that than the method using Hough transform.

## 1. Introduction

**Figure 1:**
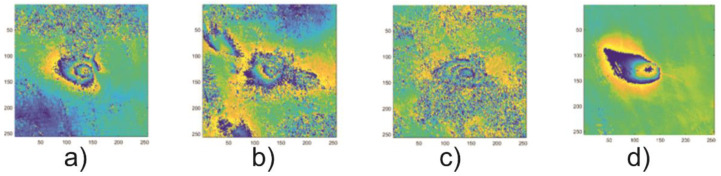
Examples of subsidence troughs visible on interferograms computed for radar images recorded on 4 April 2017 and 16 April 2017 (**a**), 16 April 2017 and 28 April 2017 (**b**). 12 December 2017 and 24 December 2017 (**c**) and 24 December 2017 and 5 January 2018 (**d**) for the area of the Upper Silesian Coal Basin, Poland (Sentinel-1A, descending).

Coal mining may lead to terrain deformation. One of the most common types of deformation is subsidence, which is indicated by vertical migration of voids in a geological medium. Subsidence can cause cracks in buildings and damage to public infrastructure such as roads, railways, gas and water pipelines, electric power lines, and sewage systems. This damage is expensive to repair and, in rare cases, it can be dangerous to human life and health (i.e., building collapse, gas explosion). Early detection of subsidence allows for preventive actions. On a small scale, this could be filling voids or reinforcing the terrain; on a large scale, it could mean moving people or infrastructure to a different location. The detection of subsidence is usually performed with geodetic or geophysical surveys, but these measurements are expensive, time-consuming, and limited only to small areas. Moreover, it is not a continuous process. Ground deformation of a large area can be monitored using differential interferometry synthetic aperture radar (DInSAR) [1], as such areas form characteristic concentric circular patterns (Figure 1). The DInSAR technique allows for the frequent detection of vertical ground deformations of large areas.

The DInSAR method is already utilized for monitoring ground deformations related to volcanic activity [2,3], earthquakes [4], or coal mining [5,6,7].

Many studies have shown the use of the DInSAR methodology to assess the ground deformation [6,7,8,9]. Over the past few years, a lot of effort has been put in the automation of DInSAR [10,11,12] or PSInsar procedures [13]. The development of an effective method of automatic detection of deformation patterns in the SAR interferograms is difficult due to the presence of noise and irregularities. Frequently occurring incomplete shapes of subsidence patterns pose yet another problem [14]. 

Detection of the subsidence in DInSAR images is based on finding any concentric circular fringes or even a single circular fringe. Therefore, the Hough transform [15] and its modifications [16,17,18,19] constitute a straightforward tool that can be used to find the subsidence area in digital images. In this method, the edge of a given image is created, and each pixel of the edge takes part in voting. The searching patterns are detected on the basis of values of accumulated votes higher than the predefined threshold value. The Hough transform [20] together with methods based on the convolution of the image with a bank of circular wavelets [12], template recognition [21], or deep convolutional neural network [22] were used for the deformation patterns detection in the SAR interferograms. None of them proved to be capable of the reliable detection of irregular circular fringes on the noisy SAR interferograms. 

Circlet transform is the recent tool of circlet detection proposed by Chauris et al. [23]. The authors show its applications in three different fields: ophthalmology, moon exploration, and coastal oceanography. The CT was also applied to detect eddies on remote sensing images of chlorophyll from the Gulf of Lion (North Western Mediterranean Sea) [24]. It was also used in medicine for the detection and counting of red blood cells in microscope blood smear images [25] or detection of the optic disc in retinal images [26]. The standard applications of CT [23] consist of the decomposition of the image into different sub-bands in order to find circular patterns. The number of circles and their radii, which are to be found in the image by the standard application of CT, are assumed a priori. There are modifications of CT that use elliptical basis functions [27] instead of the circlet basis function. The soft thresholding approach is also adopted in an iterative process, which makes it possible to detect more than one circular shape [28].

In this paper, the authors show the application of the CT method in the automatic detection of subsidence troughs in SAR interferograms designed for the monitored area. The authors present the modification of the method based on the CT coefficient analysis and a method for the automatic estimation of the radius value search range. We also show the application of the method for monitoring subsidence areas in the Upper Silesian Coal Basin.

## 2. Theoretical Background—Circlet Transform

The circlet transform (CT) [23] is a robust state-of-the-art tool for detecting objects with circular patterns in which binary image segmentation is no longer needed. The CT decomposes an image into circles called “circlets” with different radii and widths via a series of Fast Fourier Transforms (FFTs). The decomposition of the CT is formulated in the Fourier domain using special filters.

The circlet components are described by a central position (*x*_0_, *y*_0_), radius *r*_0_, and central frequency content *f*_0_. The circlet function can be written as (1) [23]:

(1) $$c_{\mu }\left(x,y\right)=\Omega \left[2\pi f_{0}\left(r-r_{0}\right)\right],$$

where $r=\sqrt{{\left(x-x_{0}\right)}^{2}+{\left(y-y_{0}\right)}^{2}}$, Ω is a fluctuating function such as a wavelet function which is formulated to reveal discontinuities. From a practical point of view, $c_{\mu }$ is defined in the 2D Fourier domain [23].

The definition of the CT is very similar to the Curvelet transform [23]. An image $f{\left(x,y\right)}_{}$ is decomposed into a sum of basic functions $c_{\mu }$

(2) $$f\left(x,y\right)=\sum_{\mu }A_{\mu }\cdot c_{\mu }\left(x,y\right).$$

In the CT, basic functions (tight frames) have a circular pattern, and the associated amplitudes $A_{\mu }$ can be obtained by (3):

(3) $$A_{\mu }=\langle f,c_{\mu }\rangle =\iint^{\;}f\left(x,y\right)\cdot c_{\mu }\left(x,y\right)dxdy.$$

From a practical point of view, the circlet’s coefficients are defined in the Fourier domain using Parseval’s theorem:

(4) $$A_{\mu }=\langle \hat{f},{\hat{c}}_{\mu }\rangle =\iint^{\;}\hat{f}\left(\omega_{1},\omega_{2}\right)\cdot {\hat{c}}_{\mu }^{*}\left(\omega_{1},\omega_{2}\right)d\omega_{1}d\omega_{2},$$

where $\hat{f}$ is the 2D Fourier transform of *f*, and ${\hat{f}}^{*}$ is the conjugate of $\hat{f}$. Since the CT is defined in the 2D Fourier domain, proper filters must be defined for the function of frequencies ${\hat{c}}_{\mu }^{*}\left(\omega_{1},\omega_{2}\right)$, the Fourier transform of *c_µ_*, such that circular shapes can be obtained for basic functions *c_µ_*(*x*,*y*) [1].

The filters are defined in the Fourier domain, and the 2D filters *G_k_* are constructed by the 1D filters *F_k_*. The *F_k_* filters are defined as (5):

(5) $$F_{k}\left(\omega \right)=\{\begin{array}{c} \begin{array}{cc} cos\left(\omega \pm \omega_{k}\right), & \left|\omega \pm \omega_{k}\right|\leq \pi /\left(N-1\right) \end{array}\\ 0,\begin{array}{cc} & otherwise \end{array} \end{array},$$

where *N* is the number of filters and $\omega_{k}=\pi \left(k-1\right)/\left(N-1\right)$. By considering the phase delay in order to obtain a circular shape in the spatial domain, the *G_k_* filters are defined as (6):

(6) $$G_{k}\left(\omega_{1},\omega_{2}\right)=e^{i\left|\omega \right|\gamma_{0}}\cdot F_{k}\left(\left|\omega \right|\right),$$

where $\omega =\left(\omega_{1},\omega_{2}\right)$ and $\left|\omega \right|=\sqrt{\omega_{1}^{2}+\omega_{2}^{2}}$. By defining the filters *G_k_*, the formulation of a circlet in the Fourier domain will be (7):

(7) $${\hat{c}}_{\mu }\left(\omega \right)=e^{i\langle \omega ,x_{c}\rangle }\cdot G_{k}\left(\omega \right),$$

where *x_c_ =* (*x*_0_, *y*_0_) is the central position and *r_0_* is the radius of the circlet. By utilizing polar coordinates, it is shown that the 2D inverse Fourier Transform of *G_k_* is circular, which indicates that the basic functions *c_µ_*(*x*, *y*) have circular shapes. 

## 3. Circlet Transform Methodology in Subsidence though Detection

**Figure 2:**
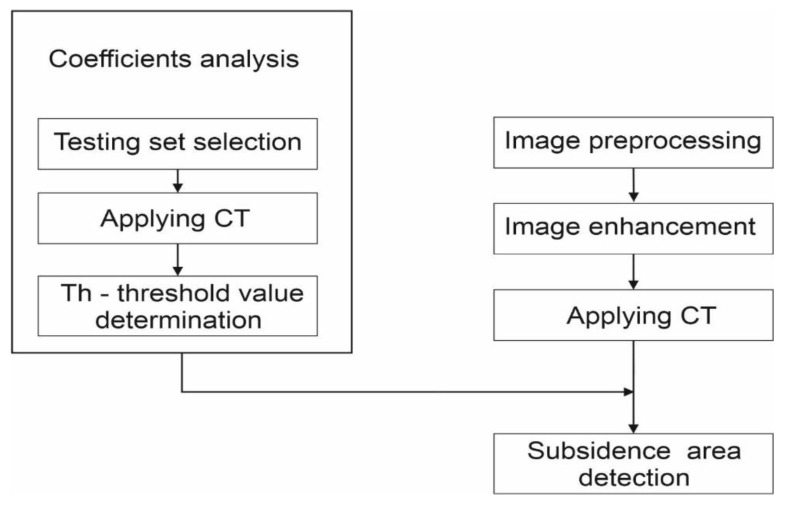
Schematic of the algorithm for the detection of subsidence troughs. The results of coefficient analysis must be taken into account during the subsidence area detection stage.

The general scheme of the subsidence trough detection algorithm is shown in Figure 2. The proposed technique consists of four main parts preceded by image pre-processing. The pre-processing is performed using the path-tracking algorithm that is commonly used to unwrap phase information. It was done in the ArcGIS program using the unwrapped mask and Goldstein algorithm. The main stages of the subsidence trough detection algorithm were made using the Mathworks Matlab 2019b computing package. The image was enhanced by histogram equalization (CLAHE). The next step—circlet transform—was directly applied to the gray-scale image. The coefficient analysis using the test set (Figure 2) is the phase of the subsidence trough detection algorithms, which is carried out only once. It is used to determine the optimal parameter value of the subsidence area detection stage. It is also the main innovation of the application of the CT results to the problem of the subsidence trough detection. The last step of the described method is the detection of subsidence area. The coefficient analysis and detection of subsidence area proposed in this work are described in detail in Section 3.1 and Section 3.3, respectively.

### 3.1. Coefficient Analysis

Detection of the single circular structure using CT is commonly performed by the selection of the highest coefficient of circlet transform (CCT) [23]. The standard approaches do not provide good results for the detection of circular shapes in noisy interferometric images. Detection based on the highest CT coefficient value is effective in the case of clear, well-formed troughs. For poorly formed troughs or troughs occurring in the parts of interferograms with signal-to-noise ratio, this approach does not produce good results. An additional drawback of detection based on the highest values of the CT coefficient is the need to specify the number of circlets to be identified. It is another parameter that depends on the area analyzed, the time when the satellite image was taken, and the atmospheric and technical conditions, which can affect the quality of the image. Thus, for this particular type of image, the method that uses coefficient analysis of CT is presented.

The coefficient analysis consisted in finding such a value of the CCT coefficient (identical for all the interferograms computed for a given area) that would allow for the detection of the maximum number of troughs possible. 

The proposed coefficient analysis could be summarized in the following steps:

- Collecting the test set containing the subsidence troughs visible on the interferograms prepared for a given area of research
- Calculating CT for the entire test set
- Determining the range of the CCT coefficient values for the test set
- Determining Th—the threshold value of CCT coefficient, $Th\in \langle CCT_{min},CCT_{max}\rangle$ the highest number of indications of subsidence troughs in the test set.

**Figure 3:**
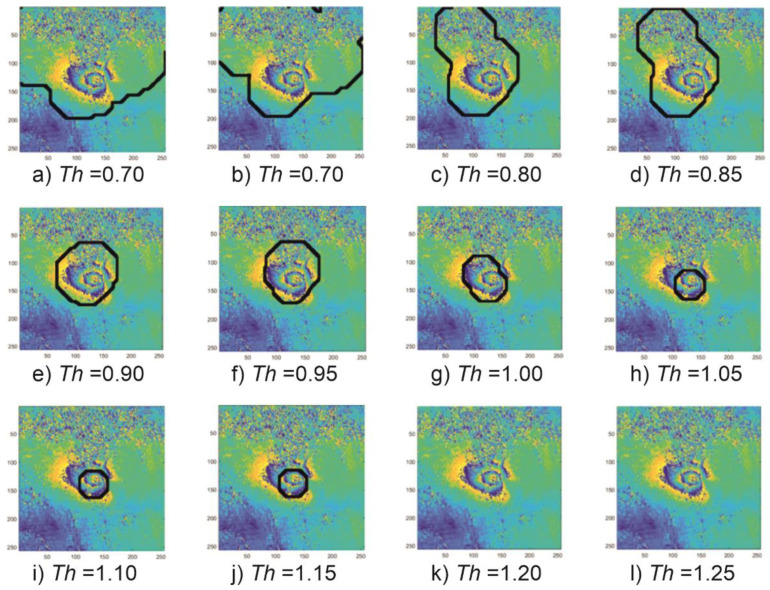
The effect of the threshold value on the effectiveness of the detection of the subsidence trough. The black outline marks the area that with the assumption of a specific *Th* value was classified as the subsidence trough.

The coefficient analysis was done based on the test set comprising 125 images of sample subsidence patterns. The patterns of 256 × 256 pixels were selected from the interferograms computed for the radar images recorded in the years 2017–2018 in the Upper Silesia Coal Basin. An example of four patterns comprising the test set is presented in Figure 1. A circlet transform was calculated for each of the 125 images. The calculations yielded the CCT coefficient of variation range for the studied areas. For the studied area of the Upper Silesian Coal Basin, the minimum value of the of the CCT coefficient module (*CCT_min_*) in the test set was 1.6 × 10^−5^. The maximum value (*CCT_max_*) calculated for that set amounted to 2.0. Figure 3 presents examples of variations of subsidence areas depending on the *Th* threshold. An example was made for the fragment of the interferogram calculated for the images taken on 4 April 2017 and 16 April 2017 (Figure 1a).

If the assumed values of the detection threshold *Th* are too small, the interferogram fragments with a large signal-to-noise ratio may be incorrectly classified as subsidence troughs (Figure 3a–d). In the CCT coefficient analysis, such ambiguities were considered errors—failure to detect a subsidence trough. On the other hand, if the assumed value of the detection threshold *Th* is too high, the trough pattern may not be detected due to the complex and irregular shape of the troughs visible in the interferograms (Figure 3k–l).

The proposed method of the CCT coefficient analysis for the Upper Silesian Coal Basin (USCB) made it possible to determine a threshold detection value that produces the best results in the classification of the subsidence troughs in the radar interferograms.

**Figure 4:**
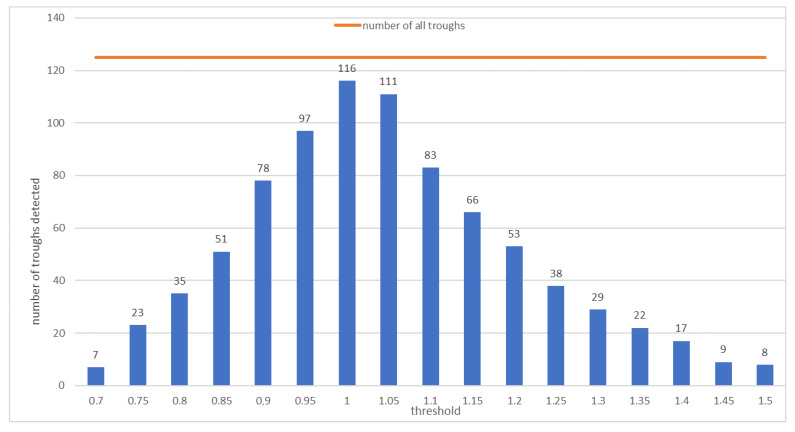
The numbers of subsidence trough detections for the Upper Silesian Coal Basin depending on the threshold *Th*. The red line indicates the number of all troughs in the test set.

Figure 4 presents a histogram showing the effectiveness of the subsidence trough detection—the number of correctly detected trough patterns depending on the assumed threshold *Th* value.

The best (91%) detection effectiveness was obtained at *Th* = 1.0. At the stage of coefficient analysis, that value was considered optimal and was used to detect the troughs in the interferograms. 

### 3.2. Comparison of the Results of Trough Detection Using CT and HT

**Figure 5:**
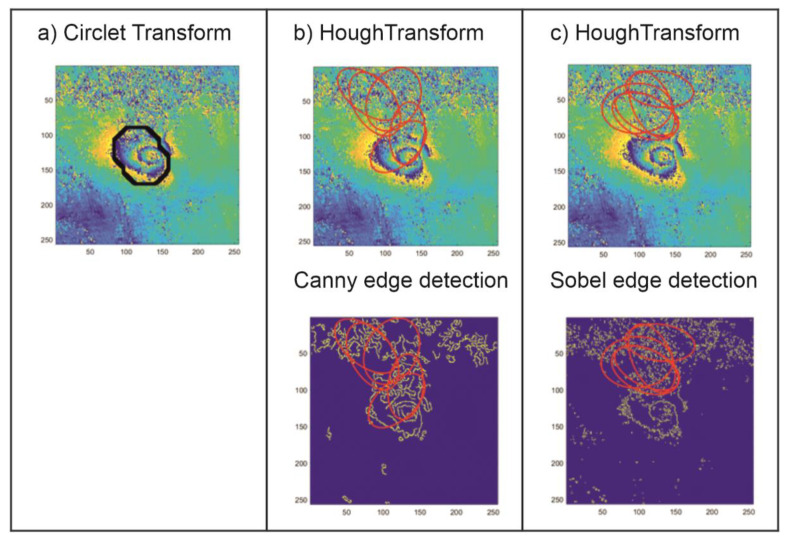
The result of the detection of a well-formed subsidence trough using the circlet transform (CT) method with *Th* = 1.0 (**a**) and the Hough transform (HT) method preceded by the edge detection using Canny (**b**) and Sobel methods (**c**).

**Figure 6:**
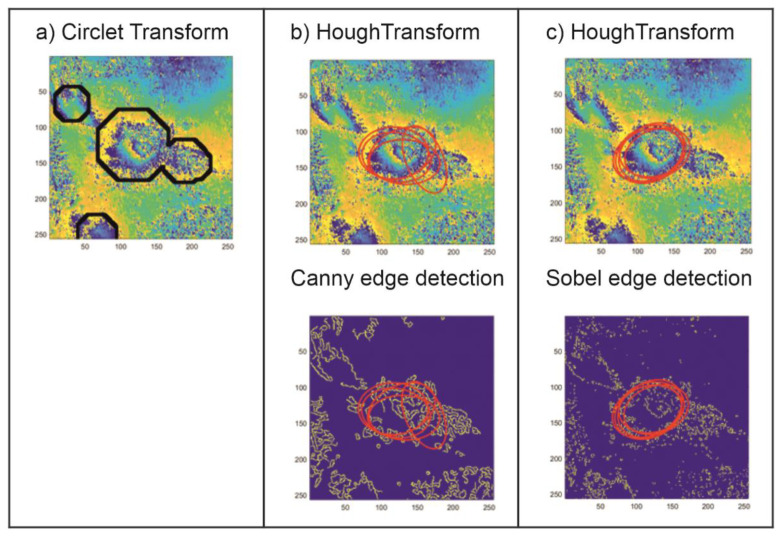
The result of the detection of four closely located subsidence troughs using the CT method with *Th* = 1.0 (**a**) and the HT method preceded by the edge detection using Canny (**b**) and Sobel methods (**c**).

**Figure 7:**
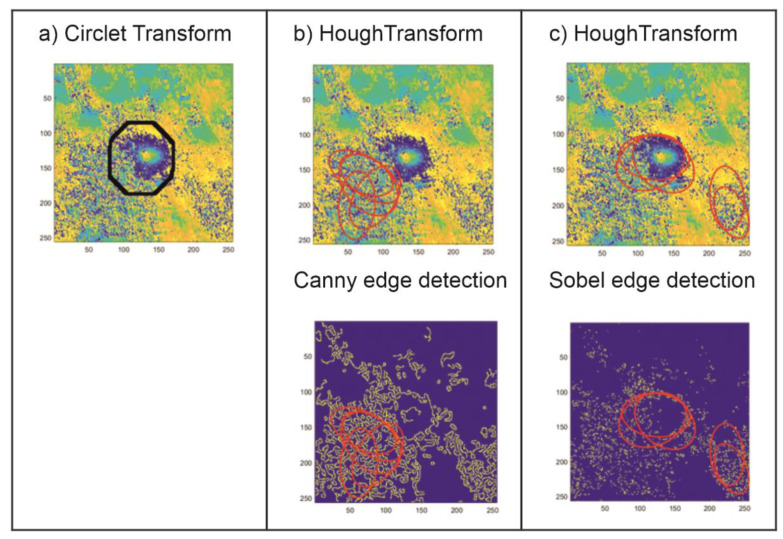
The result of the detection of a poorly formed subsidence trough using the CT method with *Th* = 1.0 (**a**) and the HT method preceded by the edge detection using Canny (**b**) and Sobel methods (**c**).

The results of the applied CCT coefficient analysis were compared with the results yielded by the traditional method used to detect circular shapes. The Hough transform (HT) method was used in an image where the edge detection methods were used; hence, its results depend strongly not only on the image noise level but also on the filtration method applied. In this study, the most popular edge detection algorithms were tested (Roberts, Sobel, Prewitt, Canny, Log, Laplacian of Gaussian). The prosed CT method, expanded by the CCT coefficient analysis, was compared with HT preceded by two types of edge detection methods: Sobel and Canny. These two methods proved to be the best at the edge detection for the test set analyzed. Figure 5, Figure 6 and Figure 7 show the comparison of the results of the CT and the HT methods for three characteristic areas: (a) a distinct trough, (b) four closely located troughs, and (c) a poorly formed subsidence trough.

The examples of subsidence trough detection presented above show that the proposed CT method with the determined threshold value, the same for the whole analayzed area, is far superior to the HT method that is commonly used to locate spherical objects. The proposed method locates subsidence troughs in noisy interferometric images (Figure 5 and Figure 7). In the case of noisy interferograms, the detection of subsidence troughs using the HT produces ambiguous results. Apart from the correct locations—TH with edge detection using Canny method (Figure 5) and Sobel (Figure 7)—it locates subsidence troughs also in very noisy interferograms. In addition, unlike the HT, the proposed CT method detects more than one subsidence trough (Figure 6).

The results of the research show that the proposed CT modification produces better results of the detection of troughs in interferometric images than the detection method based on HT. The choice of the edge detection method profoundly affects the effectiveness of the Hough algorithm.

### 3.3. Detection of the Subsidence Area

**Figure 8:**
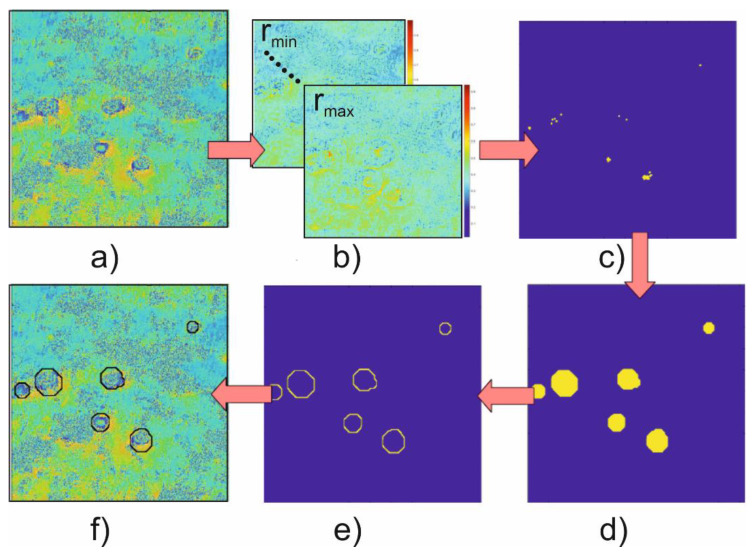
Schema of subsidence troughs detection algorithm.

Working principle of subsidence troughs detection algorithm is presented in Figure 8. For the fix parameters of the CT: *N* and *r_0_* = {*r*_1_, *r*_2_, …, *r_n_*} maps showing the fit of each radius *r*_0_ to the input data (Figure 8a) are computed (Figure 8b). Then, areas satisfying the condition CCT > *Th*, where *Th* is a threshold value obtained in coefficient analysis stage, are separated and combined into one map (Figure 8c). In the next step, for each of the points obtained as a result of the map combination, circles with radii appropriate for components map are generated (Figure 8d). The edges (Figure 8e) of the areas generated in this way indicate potential places of subsidence troughs occurrence. These edges are superimposed on the interferogram in the last step of the algorithm (Figure 8f).

## 4. Results of Subsidence Troughs Detection with CT Coefficient Analysis for Upper Silesia Coal

### 4.1. Study Area and Data Description

**Figure 9:**
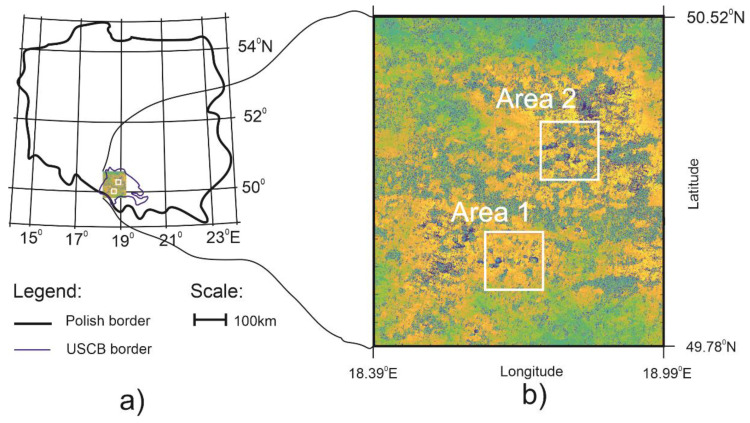
Differential, unwrapped interferogram generated for recordings performed on 10 October 2016 and 22 October 2016. It was computed from radar images of the Upper Silesia Coal Basin area, Poland (**a**). White squares on the interferogram (**b**) indicate areas with a high concentration of interferometric fringes. These two areas (Area 1 and Area 2) were selected for testing the proposed CT detection method.

The proposed algorithm was tested for the Upper Silesia Coal Basin (USCB) located in southern Poland and in the Ostrava-Karvina region in the Czech Republic. The location of the Polish part of USCB region is shown in Figure 9a. Mining activity has been conducted for over 200 years in the USCB area, which is Poland’s largest hard coal basin and one of the largest hard coal deposits in Europe. The Upper Silesian region is a big metropolitan region with 37 towns and nearly 3 million residents. It is estimated that an area of around 600 km^2^ already suffers from subsidence in USCB [29]. The proposed study is based on preprocessing differential interferograms obtained from two SAR images recorded on 10 October 2016 and 22 October 2016 from the Sentinel-1A satellite (Figure 9b).

Differential interferogram was computed by the processing of satellite SAR (synthetic aperture radar) data acquired during Sentinel-1 mission. Sentinel-1 is a polar-orbiting radar imaging system consisting of two satellites (Sentinel-1A and Sentinel-1B) in the EU Sentinels constellation. A differential interferogram was computed on the basis of SAR image pairs acquired by C-band satellite (wavelength of 18 cm) along descending orbit at an interval of twelve days but with the same illumination geometry.

The interferogram was generated using SNAP software and was filtered using the Goldstein filtering method. 

### 4.2. Results of the Subsidence Trough Detection Using CT and Compared with TH

**Figure 10:**
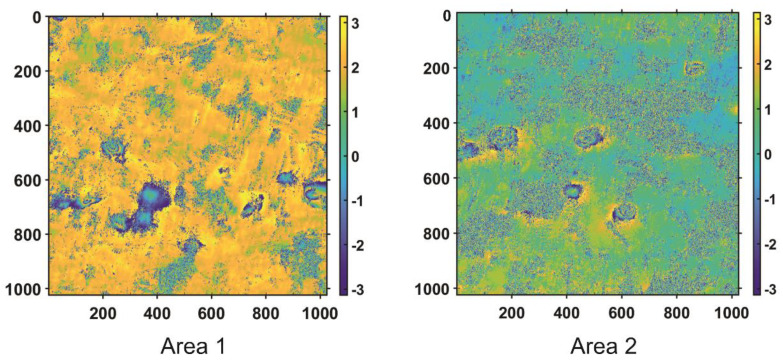
Fragments of differential interferogram generated for recordings performed on 10 October 2016 and 22 October 2016 in the Upper Silesia Coal Basin with high concentration of subsidence patterns, selected for testing the proposed CT detection method (unwrapped; polarimetric channel: VV).

The result of the subsidence troughs detection using CT and coefficients analysis is presented for two selected regions (white rectangles on Figure 9b) with a high concentration of subsidence patterns. The testing areas are presented in detail in Figure 10.

The test of the proposed subsidence troughs detection algorithm was performed for the fixed parameter values equal to *N* = 5, *Th* = 1.0 and the set of the searching radius *r_0_* = {20, 21, …, 60} pixels.

The threshold value *Th* was computed for USCB at the stage of coefficient analysis (Section 3.1). In addition, the range of searching radius was established on the basis of the size of the trough patterns in the USCB area.

For both tested areas, the authors also performed TH to find the location of circular objects connected with subsidence troughs. The Canny method with a threshold of 0.4 was used for edge detection.

**Figure 11:**
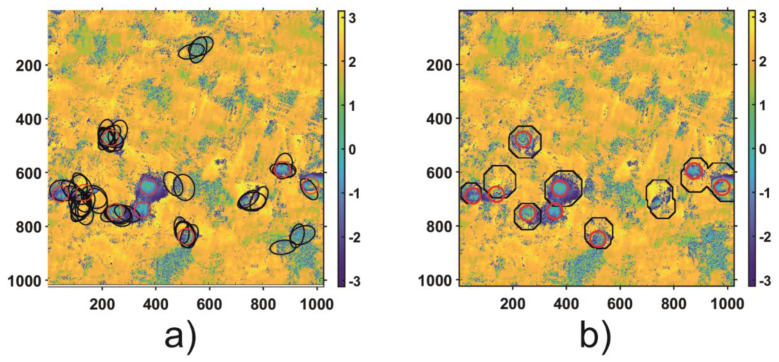
Results of subsidence trough detection using HT (black elliptical shapes on (**a**)) and CT coefficient analysis (black contours on (**b**)) computed for Area 1. The true location of subsidence troughs obtained from visual detection is depicted with red circles on both subpictures.

**Figure 12:**
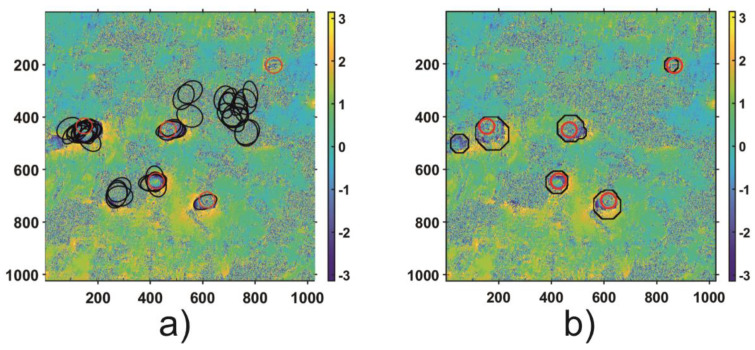
Results of subsidence trough detection using HT (black elliptical shapes on (**a**)) and CT coefficient analysis (black contours on (**b**)) computed for Area 2. The true location of subsidence troughs obtained from visual detection is depicted with red circles on both subpictures.

In order to validate the results of the automatic subsidence trough detection algorithm, both regions (Area 1 and Area 2) were thoroughly analyzed to identify visible troughs patterns. As a result of the visual analysis of both areas, nine subsidence patterns were detected in Area 1 and 5 in Area 2. The visual analysis results—the reference results—as well as the results of HT and the proposed automatic trough detection algorithm for Area 1 and Area 2 are shown in Figure 11 and Figure 12, respectively.

The results obtained confirm that detection of the troughs with the Hough algorithm does not work in the case of radar interferograms due to the high level of noise. The main problem is the edge detection, which considerably limits the effectiveness of ellipse detection. For Area 1, the analysis using TH led to the correct location of 6/9 troughs, and four areas were incorrectly located as subsidence troughs. In the case of subsidence trough detection using HT for Area 2, 4/5 troughs were located correctly. However, the number of errors amounted to 7. The results obtained by CT expanded by the CCT coefficient analysis were correct in 8/9 subsidence troughs for Area 1 and 5/5 for Area 2. What is more, for both datasets, the tested algorithm located incorrectly only one subsidence through. 

The achieved results are presented in Table 1.

On the basis of the achieved detection results—correctly detected subsidence troughs and incorrect classification of some areas as troughs—quantitative parameters were calculated that make it possible to compare both methods. The parameter specifying the error of the method connected with an incorrect area classification was calculated as the ratio of incorrect detections to the total trough number. The result analysis reveals that the effectiveness of the proposed CT method in trough detection amounts to 93%, which is 20% higher than in the case of the HT method. What is more, the CT method is characterized by very low incorrect detection values (14%), whereas the HT method has the same percentage of false detections as the percentage of correct ones—79% and 71%, respectively.

## 5. Conclusions and Further Work

This article presents the application of the circlet transform in the detection of subsidence troughs. The authors showed that this transformation can correctly detect the shapes of circles and ellipses. Preliminary results presented satisfactory detection of subsidence troughs. The problems encountered were related to not detecting incomplete or excessively flattened troughs as well as incorrect detections in noisy areas. The application of the coefficient analysis carried out after CT made it possible to reduce the number of false detections in noisy areas as compared to the CT carried out without coefficient analysis and to the HT method, which is commonly used in detection of the circular shapes.

The coefficient analysis proposed in this article can be carried out at any stage of the research. It will work for large areas as well as for a specific location if the development of the subsidence process is monitored. The proposed algorithm makes it possible to update the value of the threshold detection *Th* at any time. If the effectiveness of the algorithm decreases, it is possible to carry out the circlet coefficient analysis for a larger test set.

An additional advantage of the proposed method is the automation of the entire process realized at the subsidence detection stage. It can reduce the impact of the technician skills on the quality of the analysis.

In future research, implementation and comparison of the effectiveness of other ellipse detection algorithms are planned. Methods that use fragments of arcs in images deserve special attention because in the case of radar interferograms, subsidence troughs are very often visible only as trough fragments of elliptic arcs. The use of pre-processing (e.g., median filtering or wavelet transforms) can increase the effectiveness of the method used, as this would remove artifacts related to noise in interferograms.

## Figures and Tables

**Table 1:** Comparison of the CT and HT results in Upper Silesian Coal Basin (USCB).

Method	Detected	Undetected	Incorrect Detection
	Area 1		
HT	6	3	4
CT	8	1	1
	Area 2		
HT	4	1	7
CT	5	0	1
	Performance		
HT	71%	29%	79%
CT	93%	7%	14%

## Data Availability

The data can be accessed upon request from any of the authors.
